# Association between triglyceride glucose index and total bone mineral density: a cross-sectional study from NHANES 2011–2018

**DOI:** 10.1038/s41598-024-54192-9

**Published:** 2024-02-20

**Authors:** Ningsheng Tian, Shuai Chen, Huawei Han, Jie Jin, Zhiwei Li

**Affiliations:** Department of Orthopaedics, The Second Affiliated Hospital of Nanjing University of Chinese Medicine, No.23, Nanhu Road, Jianye District, Nanjing, 210017 Jiangsu Province People’s Republic of China

**Keywords:** Osteoporosis, BMD, TyG, Metabolism, NHANES, Endocrinology, Medical research, Risk factors

## Abstract

The Homeostatic Model Assessment for Triglyceride Glucose Index (TyG) and its related indices, including triglyceride glucose-waist circumference (TyG-WC), triglyceride glucose-waist-to-height ratio (TyG-WHtR) and triglyceride glucose-body mass index (TyG-BMI), has emerged as a practical tool for assessing insulin resistance in metabolic disorders. However, limited studies have explored the connection between TyG, TyG-related indices and osteoporosis. This population-based study, utilizing data from the National Health and Nutrition Examination Survey 2011–2018, involved 5456 participants. Through weighted multivariate linear regression and smoothed curve fitting, a significant positive correlation was found between TyG, TyG-related indices and total bone mineral density (BMD) after adjusting for covariates [β = 0.0124, 95% CI (0.0006, 0.0242), *P* = 0.0390; β = 0.0004, 95% CI (0.0003, 0.0004), *P* < 0.0001; β = 0.0116, 95% CI (0.0076, 0.0156), *P* < 0.0001; β = 0.0001, 95% CI (0.0001, 0.0001), *P* < 0.0001]. In subgroup analysis, race stratification significantly affected the relationship between TyG and total BMD. Additionally, gender and race were both significant for TyG-related indices. Non-linear relationships and threshold effects with inflection points at 9.106, 193.9265, 4.065, and 667.5304 (TyG, TyG-BMI, TyG-WHtR, TyG-WC) were identified. Saturation phenomena were observed between TyG-BMI, TyG-WC and total BMD with saturation thresholds at 314.177 and 1022.0428. These findings contributed to understanding the association between TyG, TyG-related indices and total BMD, offering insights for osteoporosis prevention and treatment.

## Introduction

Osteoporosis (OP) is a systemic degenerative skeletal disorder characterized by diminished bone mineral density (BMD) and impaired skeletal microarchitecture, leading to increased skeletal fragility and fractures^1–3^. An epidemiological investigation estimated that approximately 200 million individuals worldwide were affected by OP, which is increasing yearly^4^. As the global population continues to age, the implications of OP are expected to escalate. Projections indicate that by 2025, the direct costs associated with this condition will reach a staggering $25.3 billion annually^5^. This will inevitably place a considerable strain on both the healthcare system and the broader social economy^6^. Undoubtedly, OP has emerged as an imperative global public health concern.

As a major determinant of bone health, the measurement of BMD is a commonly used operational tool for the diagnosis of OP^7^. Relevant studies have shown that metabolic syndrome (MetS), characterized by abdominal obesity and metabolic dysfunction, has emerged as a new risk factor for BMD reduction^8^. Insulin resistance (IR) refers to the inability of peripheral tissues to respond to insulin and is the main feature of metabolic syndrome^9,10^. IR is a potential factor affecting bone density. However, current studies investigating the association between IR and OP have produced inconsistent results. Earlier study reported that the relationship between lumbar spine BMD and IR was negative^11^. In contrast, a recent research showed that IR was a protective factor for OP in a specific range^12^. There's also a evidence that reveals the connection between IR and OP risk in type 2 diabetes mellitus (T2DM) patients is significantly affected by gender^13^. A more precise comprehension of the correlation between IR and BMD could offer novel insights into the prevention and treatment of OP.

Hyperinsulinemic-euglycemic clamp (HECT) is widely considered the gold standard for evaluating IR^14^. Another prevalent method for assessing IR is the homeostasis model assessment of insulin resistance (HOMA-IR)^15^. However, both of the above two methods are limited in application scenarios, particularly because fasting insulin levels are infrequently measured in routine clinical settings and large-scale epidemiological studies. In contrast, the triglyceride glucose index (TyG) offers a simpler and more cost-effective way to assess IR. Numerous studies have shown that the accuracy of TyG is on par with that of HECT and HOMA-IR for estimating IR^16,17^. Moreover, it is imperative to note that patients with high IR often experience obesity^18^, which is also a significant factor affecting BMD^19^. In recent years, many researchers have reported that combining TyG and obesity-related indices [body mass index (BMI), waist-to-height ratio (WHtR) and waist circumference (WC)] into TyG-related indices (TyG-BMI, TyG-WHtR and TyG-WC) can enhance the ability to identify IR^20,21^. Additionally, the latest study discovered that TyG-related indices were generally more effective at predicting the progression of coronary artery calcification and identifying fatty liver than TyG^22,23^. Despite this, the evidence regarding the superiority of TyG and TyG-related indices in assessing BMD is inconclusive.

Therefore, the present study aimed to analyze the correlation between TyG, TyG-related indices and total BMD through a large-scale cross-sectional study based on the National Health and Nutrition Examination study database (NHANES).

## Methods

### Data source and study population

The NHANES is a large, ongoing cross-sectional study conducted in the U.S. designed to provide objective health statistics and address emerging public health issues among the general population (http://www.cdc.gov/nchs/nhanes/)^24^. All participants consented to the use of their data in the research, and the National Center for Health Statistics Institutional Review Board approved the survey techniques.

**Figure 1:**
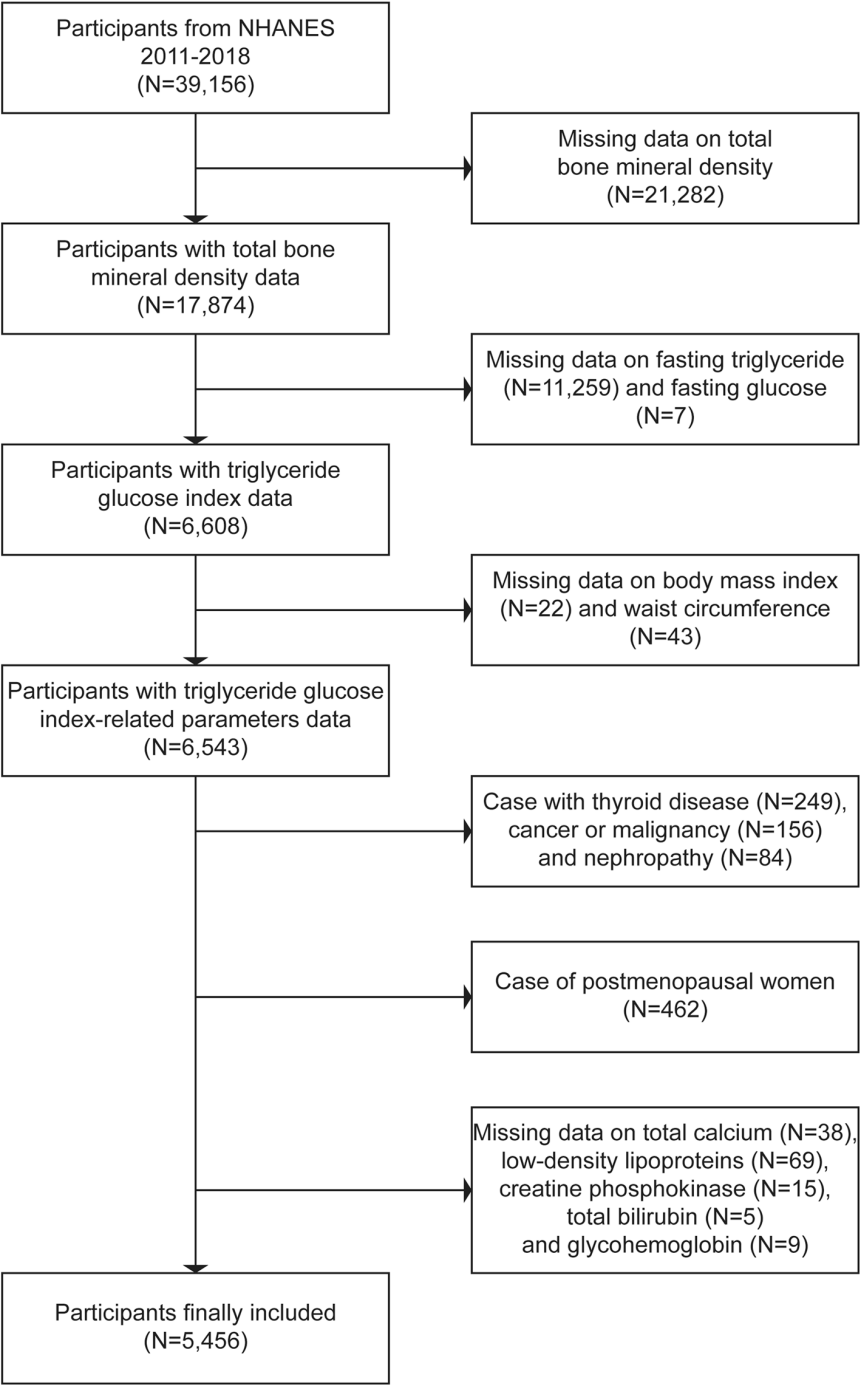
Study flowchart.

This study employed NHANES data from 2011 to 2018. In total, 39,156 participants completed the demographic survey, laboratory examination, and health condition questionnaire. The exclusion criteria were as follows: (1) Missing total BMD data (N = 21,282); (2) Missing data on fasting triglycerides (N = 11,259) and fasting glucose (N = 7); (3) Missing data on BMI (N = 22) and waist circumference (N = 43); (4) Patients with thyroid disease (N = 249), cancer or malignancy (N = 156) and nephropathy (N = 84); (5) Postmenopausal women (N = 462); (6) Missing data on total calcium (N = 38), low-density lipoproteins (N = 69), creatine phosphokinase (N = 15), total bilirubin (N = 5) and glycohemoglobin (N = 9). Ultimately, 5456 participants were enrolled in the research (Fig. 1). The accompanying NHANES variable codes and their utilization details are outlined in Supplementary Table S1.

### BMD examination

Total BMD was measured using dual-energy X-ray absorption (DXA) on a Hologic QDR 4500A fan-beam densitometer^25^. The screening tool is commonly used internationally to assess fragility-fracture risk. Radiologic technologists were certified and trained to administer DXA examinations. In the survey, DXA scans were available to all survey participants aged 8 and older, but they were not suitable for individuals weighing over 300 pounds, pregnant women, or those who had taken radiographic contrast material in the past 7 days.

### Definitions of TyG and TyG-related indices

The indices in the study were defined as follows: WHtR is defined as WC divided by body height^26^; TyG = ln [fasting triglyceride (mg/dL) × fasting glucose (mg/dL)/2]^27^; TyG-BMI = TyG × BMI^28^; TyG-WHtR = TyG × WHtR^23^; TyG-WC = TyG × WC^28^.

### Covariates

Data on demographics, examination, and laboratory are covariates. Demographics data covered age (years), gender (male and female), race/ethnicity (Mexican American, non-Hispanic white, non-Hispanic black, other race/ethnicity), level of education (less than high school, high school or equivalent, college or above) and family income-poverty ratio (family PIR). Examination data included weight (kg), height (cm), body mass index (BMI, kg/m^2^) and waist circumference (WC, cm). Finally, laboratory data consisted of fasting glucose (mg/dL), fasting triglyceride (mg/dL), alkaline phosphatase (ALP, IU/L), blood urea nitrogen (BUN, mg/dL), creatine phosphokinase (CPK, IU/L), creatinine (mg/dL), phosphorus (mg/dL), total calcium (mg/dL), uric acid (mg/dL), total bilirubin (mg/dL), glycohemoglobin (%), total cholesterol (mg/dL), high-density lipoprotein cholesterol (HDL-C, mg/dL), low-density lipoprotein cholesterol (LDL-C, mg/dL) and 25OHD2 + 25OHD3 (nmol/L).

### Statistical analysis

Statistical analysis for this study was conducted using R (http://www.r-project.org) and EmpowerStats (http://www.empowerstats.com), setting a significance level at *P* < 0.05. In accordance with NCHS analytical guidelines, we employed sample weights in all estimates to better represent the non-institutionalized civilian population in the USA. To investigate correlations between research variables and total BMD, participants were categorized into quartiles based on their total BMD values, which ranged from 0.670 to 1.728 g/cm^2^. Univariate analyses and chi-square tests were then conducted to examine these correlations. Weighted multiple linear regression analysis analyses were utilized to explore the linear associations between TyG, TyG-related indices and total BMD. The multiple linear regression analysis were structured into three distinct models: Model 1 served as the baseline and involved no adjustments for any variables; Model 2 included adjustments for age, gender and race/ethnicity; Model 3 adjusted for a variety of factors, including age, gender, race/ethnicity, level of education, family PIR, ALP, BUN, CPK, creatinine, phosphorus, total calcium, uric acid, total bilirubin, glycohemoglobin, total cholesterol, HDL-C, LDL-C and 25OHD2 + 25OHD3. Subgroup analyses were also conducted, focusing specifically on variations across gender and race. In the final stage of our statistical analysis, smoothing curve fitting techniques and threshold effects evaluations were employed to assess any non-linear relationships between TyG, TyG-related indices and total BMD respectively.

### Ethics approval and consent to participate

All procedures performed in studies involving human participants were in accordance with the ethical standards of the institutional and/or national research committee and with the 1964 Helsinki declaration and its later amendments or comparable ethical standards. All analyses were based on data of the National Health and Nutrition Examination Survey (NHANES). The study was approved by the ethics review board of the National Center for Health Statistics. The detailed information located on the NHANES website. Written informed consent was obtained from each participant before their inclusion on the NHANES database. Detailed information on the ethics application and written informed consent are provided on the NHANES website.

## Results

### Baseline characteristics

After applying inclusion and exclusion criteria, 5456 participants were included in the study with a mean age of 30.33 ± 13.55 years. The sample comprised 55.65% males and 44.35% females, along with 17.19% Mexican–American people, 32.04% non-Hispanic white people, 21.13% non-Hispanic black people and 29.64% individuals from other racial backgrounds.

As shown in Table 1, the clinical characteristics of the participants are listed in stratified groups based on the four categories of total BMD. Compared to the bottom quartile, those in the top total BMD quartile were more likely to be male and older, had a higher proportion of non-Hispanic white and non-Hispanic black, exhibited a higher PIR and education level, and showed increased levels of TyG, TyG-BMI, TyG-WHtR, TyG-WC, weight, height, BMI, WC, fasting glucose, fasting triglyceride, BUN, CPK, creatinine, uric acid, total bilirubin, glycohemoglobin, total cholesterol, LDL-C and 25OHD2 + 25OHD3. Conversely, they demonstrated lower levels of ALP, phosphorus, total calcium and HDL-C (*P* < 0.05).

**Table 1:** Basic characteristics of the research population based on total BMD quartiles.

Total BMD (g/cm^2^)	Quartile 1 (0.670–1.018)	Quartile 2 (1.019–1.094)	Quartile 3 (1.095–1.167)	Quartile 4 (1.168–1.728)	*P* value
Age (years)	26.81 ± 14.63	32.21 ± 12.96	34.08 ± 12.22	35.92 ± 11.82	< 0.0001
Gender (%)					< 0.0001
Male	44.19	47.20	57.24	76.36	
Female	55.81	52.80	42.76	23.64	
Race/ethnicity (%)					< 0.0001
Mexican American	15.40	13.09	12.02	8.94	
Non-Hispanic White	56.08	59.23	61.18	57.98	
Non-Hispanic Black	6.87	7.11	10.27	19.82	
Other race/ethnicity	21.64	20.57	16.53	13.26	
Education level (%)					< 0.0001
Less than high school	50.56	28.58	21.52	17.38	
High school or equivalent	12.72	17.78	22.94	21.59	
College or above	36.72	53.64	55.54	61.03	
Family PIR	2.56 ± 1.64	2.55 ± 1.64	2.86 ± 1.69	2.97 ± 1.64	< 0.0001
Weight (kg)	65.93 ± 18.85	77.93 ± 21.39	81.68 ± 19.86	88.45 ± 20.00	< 0.0001
Height (cm)	163.44 ± 9.15	167.61 ± 8.53	170.36 ± 8.94	174.12 ± 7.95	< 0.0001
BMI (kg/cm^2^)	24.52 ± 6.13	27.69 ± 7.13	28.16 ± 6.65	29.15 ± 6.22	< 0.0001
WC (cm)	86.07 ± 16.63	93.84 ± 17.40	95.40 ± 16.58	98.19 ± 15.77	< 0.0001
Fasting glucose (mg/dL)	97.98 ± 14.60	100.44 ± 22.06	100.69 ± 22.75	104.99 ± 34.19	< 0.0001
Fasting triglyceride (mg/dL)	92.56 ± 57.73	103.02 ± 62.96	101.87 ± 63.33	105.12 ± 65.01	< 0.0001
ALP (IU/L)	117.46 ± 91.24	74.95 ± 43.07	68.87 ± 28.77	65.57 ± 23.92	< 0.0001
BUN (mg/dL)	11.30 ± 3.39	11.80 ± 3.56	12.36 ± 3.70	13.28 ± 4.18	< 0.0001
CPK (IU/L)	121.48 ± 120.75	135.81 ± 128.26	161.84 ± 311.36	208.20 ± 327.07	< 0.0001
Creatinine (mg/dL)	0.71 ± 0.16	0.80 ± 0.17	0.84 ± 0.17	0.92 ± 0.18	< 0.0001
Phosphorus (mg/dL)	4.05 ± 0.74	3.76 ± 0.59	3.67 ± 0.55	3.63 ± 0.55	< 0.0001
Total calcium (mg/dL)	9.44 ± 0.42	9.38 ± 0.35	9.35 ± 0.32	9.35 ± 0.33	< 0.0001
Uric acid (mg/dL)	4.96 ± 1.21	5.20 ± 1.25	5.44 ± 1.35	5.72 ± 1.35	< 0.0001
Total bilirubin (mg/dL)	0.62 ± 0.30	0.63 ± 0.32	0.67 ± 0.35	0.67 ± 0.34	< 0.0001
Glycohemoglobin (%)	5.33 ± 0.53	5.37 ± 0.70	5.39 ± 0.74	5.51 ± 1.03	< 0.0001
Total cholesterol (mg/dL)	173.32 ± 37.93	180.02 ± 39.14	181.93 ± 38.24	181.41 ± 36.04	< 0.0001
HDL-C (mg/dL)	54.65 ± 13.56	52.11 ± 13.42	52.82 ± 15.42	51.09 ± 13.21	< 0.0001
LDL-C (mg/dL)	100.18 ± 33.35	107.29 ± 34.14	108.73 ± 32.99	109.30 ± 32.27	< 0.0001
25OHD2 + 25OHD3 (nmol/L)	62.61 ± 22.47	63.49 ± 23.24	64.63 ± 24.00	65.60 ± 24.85	0.0075
TyG	8.24 ± 0.62	8.37 ± 0.63	8.36 ± 0.63	8.42 ± 0.65	< 0.0001
TyG-BMI	203.71 ± 59.66	233.28 ± 68.14	237.00 ± 65.09	246.60 ± 60.64	< 0.0001
TyG-WHtR	4.37 ± 1.01	4.72 ± 1.05	4.72 ± 1.04	4.77 ± 0.96	< 0.0001
TyG-WC	714.47 ± 172.34	790.26 ± 180.75	802.56 ± 176.23	830.79 ± 167.95	< 0.0001

Mean ± SD for continuous variables: the *P* value was calculated by the weighted linear regression model. (percentage) for categorical variables: the *P* value was calculated by the weighted chi-square test.

*Family PIR* the ratio of family income to poverty, *BMI* body mass index, *WC* waist circumference, *ALP* alkaline phosphatase, *BUN* blood urea nitrogen, *CPK* creatine phosphokinase, *HDL-C* high-density lipoprotein cholesterol, *LDL-C* low-density lipoprotein cholesterol, *TyG* triglyceride glucose index, *TyG-BMI* triglyceride glucose-body mass index, *TyG-WHtR* triglyceride glucose-waist-to-height ratio, *TyG-WC* triglyceride glucose-waist circumference.

### Association between TyG, TyG-related indices and total BMD

To investigate the relationships between TyG, TyG-related indices and total BMD, multiple linear regression models ware used. Table 2 presents the outcomes for three distinct models. All models exhibited a positive correlation between TyG, TyG-related indices and total BMD, TyG-BMI, TyG-WHtR and TyG-WC demonstrated a statistically correlated with total BMD (*P* < 0.0001). After adjusting for all confounding variables, one-unit increments in TyG, TyG-BMI, TyG-WHtR and TyG-WC were linked with 0.0124 g/cm^2^, 0.0004 g/cm^2^, 0.0116 g/cm^2^, and 0.0001 g/cm^2^ increase in total BMD, respectively. When TyG-BMI, TyG-WHtR and TyG-WC were grouped into quartiles, this association persisted statistically significant (*P* < 0.0001).

**Table 2:** Association between TyG, TyG-BMI, TyG-WHtR, TYG-WC and total BMD.

Exposure	Model 1 β (95% CI), *P* value	Model 2 β (95% CI), *P* value	Model 3 β (95% CI), *P* value
TyG (continuous)	0.0176 (0.0130, 0.0222) < 0.0001	0.0016 (− 0.0031, 0.0063) 0.4987	0.0124 (0.0006, 0.0242) 0.0390
TyG (quartile)			
Q1	Reference	Reference	Reference
Q2	0.0159 (0.0072, 0.0245) 0.0003	0.0112 (0.0031, 0.0192) 0.0065	0.0017 (− 0.0062, 0.0095) 0.6766
Q3	0.0282 (0.0197, 0.0366) < 0.0001	0.0153 (0.0073, 0.0233) 0.0002	0.0045 (− 0.0044, 0.0134) 0.3243
Q4	0.0255 (0.0170, 0.0339) < 0.0001	− 0.0019 (− 0.0103, 0.0065) 0.6587	− 0.0130 (− 0.0268, 0.0008) 0.0648
TyG-BMI (continuous)	0.0004 (0.0004, 0.0005) < 0.0001	0.0003 (0.0003, 0.0003) < 0.0001	0.0004 (0.0003, 0.0004) < 0.0001
TyG-BMI (quartile)			
Q1	Reference	Reference	Reference
Q2	0.0665 (0.0582, 0.0748) < 0.0001	0.0554 (0.0475, 0.0634) < 0.0001	0.0376 (0.0298, 0.0455) < 0.0001
Q3	0.0848 (0.0766, 0.0930) < 0.0001	0.0633 (0.0551, 0.0715) < 0.0001	0.0539 (0.0452, 0.0627) < 0.0001
Q4	0.0883 (0.0801, 0.0965) < 0.0001	0.0678 (0.0596, 0.0761) < 0.0001	0.0702 (0.0602, 0.0802) < 0.0001
TyG-WHTR (continuous)	0.0155 (0.0126, 0.0183) < 0.0001	0.0080 (0.0051, 0.0109) < 0.0001	0.0116 (0.0076, 0.0156) < 0.0001
TyG-WHTR (quartile)			
Q1	Reference	Reference	Reference
Q2	0.0466 (0.0380, 0.0552) < 0.0001	0.0402 (0.0320, 0.0483) < 0.0001	0.0274 (0.0195, 0.0354) < 0.0001
Q3	0.0525 (0.0441, 0.0608) < 0.0001	0.0327 (0.0243, 0.0410) < 0.0001	0.0272 (0.0182, 0.0363) < 0.0001
Q4	0.0539 (0.0454, 0.0623) < 0.0001	0.0332 (0.0246, 0.0418) < 0.0001	0.0347 (0.0239, 0.0455) < 0.0001
TyG-WC (continuous)	0.0001 (0.0001, 0.0002) < 0.0001	0.0001 (0.0001, 0.0001) < 0.0001	0.0001 (0.0001, 0.0001) < 0.0001
TyG-WC (quartile)			
Q1	Reference	Reference	Reference
Q2	0.0632 (0.0547, 0.0718) < 0.0001	0.0523 (0.0441, 0.0606) < 0.0001	0.0346 (0.0264, 0.0427) < 0.0001
Q3	0.0756 (0.0673, 0.0839) < 0.0001	0.0537 (0.0452, 0.0622) < 0.0001	0.0470 (0.0378, 0.0562) < 0.0001
Q4	0.0843 (0.0760, 0.0925) < 0.0001	0.0550 (0.0463, 0.0638) < 0.0001	0.0567 (0.0457, 0.0677) < 0.0001

Model 1: no covariates were adjusted. Model 2: age, gender, and race were adjusted. Model 3: age, gender, race, education level, family PIR, ALP, BUN, CPK, creatinine, phosphorus, total calcium, uric acid, total bilirubin, glycohemoglobin, total cholesterol, HDL-C, LDL-C and 25OHD2 + 25OHD3 were adjusted.

### Subgroup analysis

A subgroup analysis was conducted to evaluate the stability of the relationship between TyG, TyG-related indices and total BMD across various demographic contexts. As illustrated in Tables 3, 4, 5 and 6, positive correlations between TyG-related indices and total BMD were significantly affected by gender stratification (*P* < 0.05 for interactions). Racial differences in absolute effect sizes were statistically significant in racially stratified subgroup analyses of TyG and TyG-related indices (*P* < 0.05 for interactions). Furthermore, positive correlations between TyG-BMI, TyG-WC and total BMD were significant not only unadjusted but also fully adjusted (*P* < 0.0001).

**Table 3:** Subgroup analysis between TyG and total BMD.

	Model 1 β (95% CI), *P* value	Model 2 β (95% CI), *P* value	Model 3 β (95% CI), *P* value
Stratified by gender			
Male	0.0140 (0.0077, 0.0203) < 0.0001	0.0047 (− 0.0019, 0.0113) 0.1622	0.0101 (− 0.0055, 0.0256) 0.2049
Female	− 0.0005 (− 0.0073, 0.0062) 0.8730	− 0.0030 (− 0.0095, 0.0035) 0.3710	0.0182 (0.0006, 0.0357) 0.0430
*P* for interaction	0.0001	0.0504	0.3478
Stratified by race			
Mexican American	0.0301 (0.0199, 0.0404) < 0.0001	0.0075 (− 0.0032, 0.0181) 0.1688	0.0366 (0.0090, 0.0642) 0.0096
Non-Hispanic White	0.0225 (0.0145, 0.0305) < 0.0001	0.0012 (− 0.0071, 0.0094) 0.7834	0.0123 (− 0.0080, 0.0326) 0.2368
Non-Hispanic Black	0.0337 (0.0228, 0.0445) < 0.0001	0.0161 (0.0046, 0.0277) 0.0064	0.0298 (0.0054, 0.0542) 0.0168
Other race/ethnicity	0.0139 (0.0056, 0.0223) 0.0011	− 0.0111 (− 0.0196, − 0.0027) 0.0095	− 0.0056 (− 0.0265, 0.0154) 0.6031
*P* for interaction	0.0054	0.0244	0.0198

Model 1: no covariates were adjusted. Model 2: age, gender and race were adjusted. Model 3: age, gender, race, education level, family PIR, ALP, BUN, CPK, creatinine, phosphorus, total calcium, uric acid, total bilirubin, glycohemoglobin, total cholesterol, HDL-C, LDL-C and 25OHD2 + 25OHD3 were adjusted.

**Table 4:** Subgroup analysis between TyG-BMI and total BMD.

	Model 1 β (95% CI), *P* value	Model 2 β (95% CI), *P* value	Model 3 β (95% CI), *P* value
Stratified by gender			
Male	0.0004 (0.0004, 0.0005) < 0.0001	0.0004 (0.0003, 0.0004) < 0.0001	0.0003 (0.0002, 0.0004) < 0.0001
Female	0.0003 (0.0003, 0.0004) < 0.0001	0.0002 (0.0002, 0.0003) < 0.0001	0.0004 (0.0003, 0.0005) < 0.0001
*P* for interaction	0.0433	0.0139	0.0741
Stratified by race			
Mexican American	0.0005 (0.0004, 0.0006) < 0.0001	0.0004 (0.0003, 0.0005) < 0.0001	0.0004 (0.0003, 0.0006) < 0.0001
Non-Hispanic White	0.0004 (0.0003, 0.0005) < 0.0001	0.0003 (0.0002, 0.0003) < 0.0001	0.0003 (0.0002, 0.0004) < 0.0001
Non-Hispanic Black	0.0004 (0.0003, 0.0005) < 0.0001	0.0004 (0.0002, 0.0005) < 0.0001	0.0003 (0.0002, 0.0004) < 0.0001
Other race/ethnicity	0.0005 (0.0004, 0.0006) < 0.0001	0.0003 (0.0002, 0.0004) < 0.0001	0.0005 (0.0004, 0.0006) < 0.0001
*P* for interaction	0.1024	0.0249	0.0002

Model 1: no covariates were adjusted. Model 2: age, gender and race were adjusted. Model 3: age, gender, race, education level, family PIR, ALP, BUN, CPK, creatinine, phosphorus, total calcium, uric acid, total bilirubin, glycohemoglobin, total cholesterol, HDL-C, LDL-C and 25OHD2 + 25OHD3 were adjusted.

**Table 5:** Subgroup analysis between TyG-WHtR and total BMD.

	Model 1 β (95% CI), *P* value	Model 2 β (95% CI), *P* value	Model 3 β (95% CI), *P* value
Stratified by gender			
Male	0.0149 (0.0108, 0.0190) < 0.0001	0.0076 (0.0032, 0.0120) 0.0008	0.0036 (− 0.0019, 0.0091) 0.1962
Female	0.0147 (0.0111, 0.0182) < 0.0001	0.0083 (0.0047, 0.0119) < 0.0001	0.0191 (0.0141, 0.0241) < 0.0001
*P* for interaction	0.9866	0.7375	0.0002
Stratified by race			
Mexican American	0.0216 (0.0151, 0.0281) < 0.0001	0.0124 (0.0056, 0.0193) < 0.0001	0.0192 (0.0103, 0.0282) < 0.0001
Non-Hispanic White	0.0157 (0.0109, 0.0206) < 0.0001	0.0060 (0.0010, 0.0110) 0.0184	0.0048 (− 0.0018, 0.0114) 0.1557
Non-Hispanic Black	0.0174 (0.0109, 0.0240) < 0.0001	0.0128 (0.0058, 0.0198) 0.0004	0.0117 (0.0032, 0.0202) 0.0070
Other race/ethnicity	0.0183 (0.0128, 0.0238) < 0.0001	0.0077 (0.0022, 0.0133) 0.0066	0.0232 (0.0160, 0.0304) < 0.0001
*P* for interaction	0.4474	0.4230	0.0036

Model 1: no covariates were adjusted. Model 2: age, gender and race were adjusted. Model 3: age, gender, race, education level, family PIR, ALP, BUN, CPK, creatinine, phosphorus, total calcium, uric acid, total bilirubin, glycohemoglobin, total cholesterol, HDL-C, LDL-C and 25OHD2 + 25OHD3 were adjusted.

**Table 6:** Subgroup analysis between TyG-WC and total BMD.

	Model 1 β (95% CI), *P* value	Model 2 β (95% CI), *P* value	Model 3 β (95% CI), *P* value
Stratified by gender			
Male	0.0001 (0.0001, 0.0002) < 0.0001	0.0001 (0.0001, 0.0001) < 0.0001	0.0001 (0.0001, 0.0001) < 0.0001
Female	0.0001 (0.0001, 0.0001) < 0.0001	0.0001 (0.0001, 0.0001) < 0.0001	0.0002 (0.0001, 0.0002) < 0.0001
*P* for interaction	0.5747	0.2585	0.0027
Stratified by race			
Mexican American	0.0002 (0.0002, 0.0002) < 0.0001	0.0001 (0.0001, 0.0002) < 0.0001	0.0002 (0.0001, 0.0002) < 0.0001
Non-Hispanic White	0.0001 (0.0001, 0.0002) < 0.0001	0.0001 (0.0000, 0.0001) < 0.0001	0.0001 (0.0000, 0.0001) < 0.0001
Non-Hispanic Black	0.0001 (0.0001, 0.0002) < 0.0001	0.0001 (0.0001, 0.0001) < 0.0001	0.0001 (0.0001, 0.0001) < 0.0001
Other race/ethnicity	0.0002 (0.0001, 0.0002) < 0.0001	0.0001 (0.0001, 0.0001) < 0.0001	0.0002 (0.0001, 0.0002) < 0.0001
*P* for interaction	0.995	0.0260	0.0002

Model 1: no covariates were adjusted. Model 2: age, gender and race were adjusted. Model 3: age, gender, race, education level, family PIR, ALP, BUN, CPK, creatinine, phosphorus, total calcium, uric acid, total bilirubin, glycohemoglobin, total cholesterol, HDL-C, LDL-C and 25OHD2 + 25OHD3 were adjusted.

### Non-linearity and saturation effect analysis between TyG, TyG-related indices and total BMD

**Figure 2:**
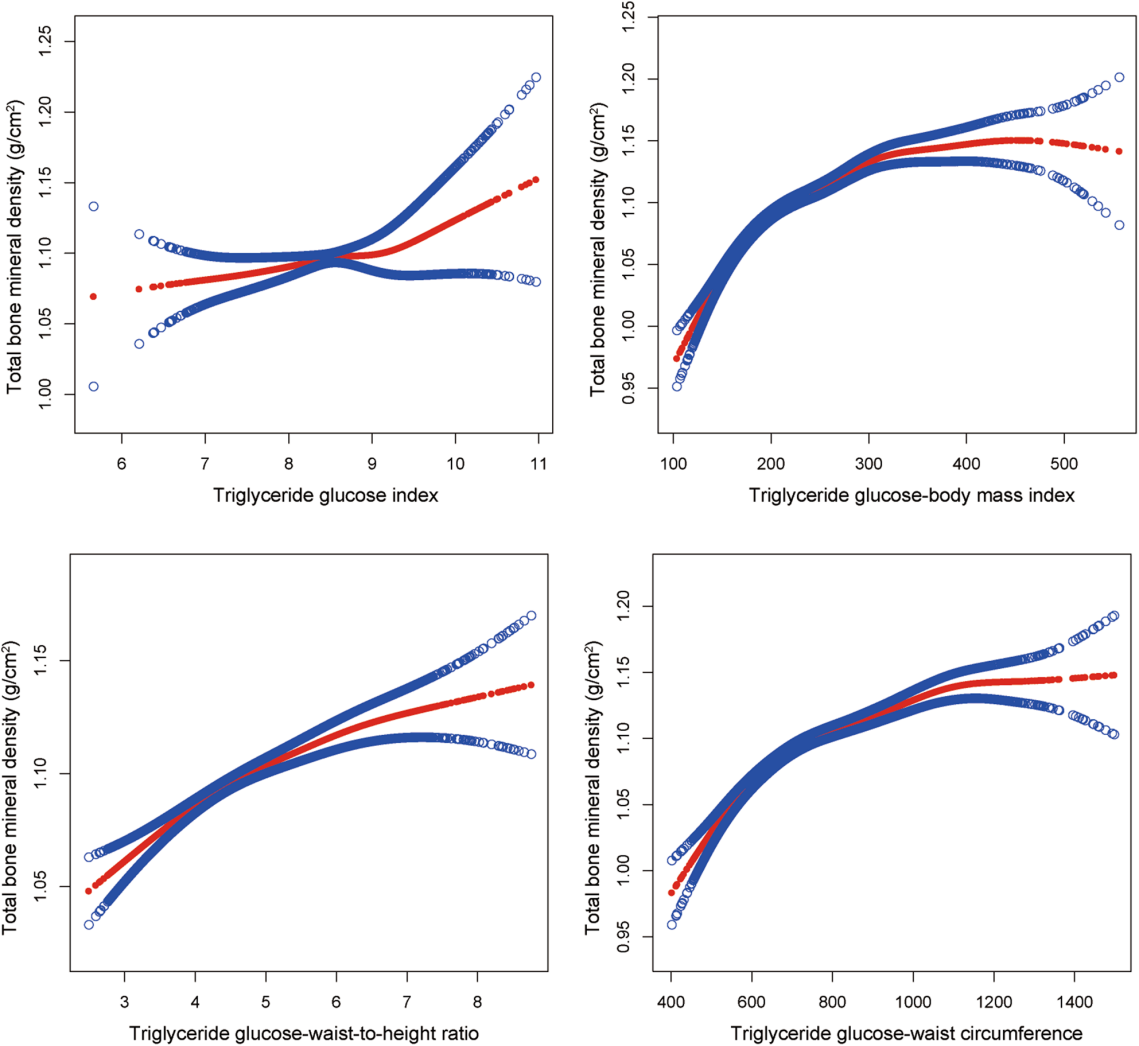
Association between TyG, TyG-related indices and total BMD.  Solid red line represents the smooth curve fit between variables. Blue bands represent the 95% confidence interval from the fit. Gender, race, age, education level, family PIR, ALP, BUN, CPK, creatinine, phosphorus, total calcium, uric acid, total bilirubin, glycohemoglobin, total cholesterol, HDL-C, LDL-C and 25OHD2 + 25OHD3 were adjusted.

As a result of smooth curve fitting, the non-linear association and saturation phenomenon between TyG, TyG-related indices, and total BMD have been delineated (Fig. 2). For TyG, TyG-BMI, TyG-WHtR and TyG-WC, the thresholds were 9.106, 193.9265, 4.065 and 667.5304, respectively (Tables 7, 8, 9, 10). The regression coefficient increases when TyG exceeds the threshold. By contrast, when TyG-BMI, TyG-WHtR and TyG-WC exceed their thresholds, the regression coefficient decreases accordingly. Furthermore, TyG-BMI and TyG-WC displayed saturation effects on total BMD, with saturation thresholds of 314.177 and 1022.0428, respectively.

**Table 7:** Saturation effect analysis of TyG on total BMD.

Total BMD	β (95% CI)	*P* value
TyG		
Model I	0.0124 (0.0006, 0.0242)	0.0390
Model II		
Inflection point (K)	9.106	
< K point effect 1	0.0209 (0.0084, 0.0334)	0.0010
> K point effect 2	0.0752 (0.0422, 0.1083)	< 0.0001
Effect 2 minus effect1	0.0543 (0.0276, 0.0811)	< 0.0001
Predicted value of the equation at the folding point	1.1177 (1.1116, 1.1238)	
Log-likelihood ratio test		< 0.0001

Age, gender, race, education level, family PIR, ALP, BUN, CPK, creatinine, phosphorus, total calcium, uric acid, total bilirubin, glycohemoglobin, total cholesterol, HDL-C, LDL-C and 25OHD2 + 25OHD3 were adjusted.

Model I, univariate linear regression; Model II, two-piecewise regression model.

**Table 8:** Saturation effect analysis of TyG-BMI on total BMD.

Total BMD	β (95% CI)	*P* value
TyG-BMI		
Model I	0.0004 (0.0003, 0.0004)	< 0.0001
Model II		
Inflection point (K 1)	193.9265	
< K point effect 1	0.0013 (0.0011, 0.0015)	< 0.0001
> K point effect 2	0.0002 (0.0002, 0.0003)	< 0.0001
Effect 2 minus effect1	− 0.0011 (− 0.0013, − 0.0009)	< 0.0001
Predicted value of the equation at the folding point	1.1222 (1.1174, 1.1270)	
Log-likelihood ratio test		< 0.0001
Inflection point (K 2)	314.177	
< K point effect 1	0.0004 (0.0003, 0.0006)	< 0.0001
> K point effect 2	0.0000 (− 0.0001, 0.0002)	0.6523
Effect 2 minus effect1	− 0.0004 (− 0.0007, − 0.0001)	0.0025
Predicted value of the equation at the folding point	1.1386 (1.1294, 1.1479)	
Log-likelihood ratio test		0.0020

Age, gender, race, education level, family PIR, ALP, BUN, CPK, creatinine, phosphorus, total calcium, uric acid, total bilirubin, glycohemoglobin, total cholesterol, HDL-C, LDL-C and 25OHD2 + 25OHD3 were adjusted.

Model I, univariate linear regression; Model II, two-piecewise regression model.

**Table 9:** Saturation effect analysis of TyG-WHtR on total BMD.

Total BMD	β (95% CI)	*P* value
TyG-WHtR		
Model I	0.0116 (0.0076, 0.0156)	< 0.0001
Model II		
Inflection point (K)	4.065	
< K point effect 1	0.0408 (0.0291, 0.0524)	< 0.0001
> K point effect 2	0.0062 (0.0017, 0.0106)	0.0065
Effect 2 minus effect1	− 0.0346 (− 0.0476, − 0.0216)	< 0.0001
Predicted value of the equation at the folding point	1.1169 (1.1117, 1.1221)	
Log-likelihood ratio test		< 0.0001

Age, gender, race, education level, family PIR, ALP, BUN, CPK, creatinine, phosphorus, total calcium, uric acid, total bilirubin, glycohemoglobin, total cholesterol, HDL-C, LDL-C and 25OHD2 + 25OHD3 were adjusted.

Model I, univariate linear regression; Model II, two-piecewise regression model.

**Table 10:** Saturation effect analysis of TyG-WC on total BMD.

Total BMD	β (95% CI)	*P* value
TyG-WC		
Model I	0.0001 (0.0001, 0.0001)	< 0.0001
Model II		
Inflection point (K 1)	667.5304	
< K point effect 1	0.0004 (0.0003, 0.0005)	< 0.0001
> K point effect 2	0.0001 (0.0001, 0.0001)	< 0.0001
Effect 2 minus effect1	− 0.0003 (− 0.0004, − 0.0002)	< 0.0001
Predicted value of the equation at the folding point	1.1140 (1.1090, 1.1191)	
Log-likelihood ratio test		< 0.0001
Inflection point (K 2)	1022.0428	
< K point effect 1	0.0001 (0.0001, 0.0002)	< 0.0001
> K point effect 2	0.0000 (− 0.0001, 0.0001)	0.8095
Effect 2 minus effect1	− 0.0001 (− 0.0002, − 0.0001)	< 0.0001
Predicted value of the equation at the folding point	1.1562 (1.1500, 1.1625)	
Log-likelihood ratio test		< 0.0001

Age, gender, race, education level, family PIR, ALP, BUN, CPK, creatinine, phosphorus, total calcium, uric acid, total bilirubin, glycohemoglobin, total cholesterol, HDL-C, LDL-C and 25OHD2 + 25OHD3 were adjusted.

Model I, univariate linear regression; Model II, two-piecewise regression model.

### Supplementary analysis

To ensure the stability of the TyG and TyG-related indices data in NHANES, we performed supplementary correlation analyses between TyG and TyG-related indices and HOMA-IR as well as fasting insulin. The results revealed a positive correlation between TyG and TyG-related indices with both HOMA-IR and fasting insulin. This observation aligns with the findings of previous studies, affirming the consistency of our results with established research in the field (Supplementary Table S2–S3).

To ensure the relative stability of experimental results, missing data for Family PIR were retained, introducing potential bias in the outcomes of Model 3. To address this potential bias, additional subgroup analyses were conducted using all covariates except Family PIR (Supplementary Table S4–S7). In this subgroup analysis, no data were missing in the study population, revealing only a minor deviation compared to Model 3, and indicating no substantive impact on the experimental conclusions.

## Discussion

To our knowledge, this is the first cross-sectional study in the United States to examine correlations between TyG, TyG-related indices and BMD, revealing the close relationship between IR and bone metabolism. It was found that IR evaluated by TyG and TyG-related indices was significantly positively correlated with BMD among Americans. This is consistent with previous studies, such as an ongoing prospective study that found TyG-BMI was positively related to BMD and geometry in non-diabetic middle-aged and elderly Chinese individuals^29^. Moreover, a single-center retrospective study discovered that IR was positively correlated with BMD in postmenopausal women, as well as a protective factor for OP^12^. As per another study, higher IR values were associated with higher BMD values in non-diabetic older adults^30^.

Currently, the underlying mechanisms that explain the observed positive correlation between TyG, TyG-related indices and total BMD remain incompletely understood. Several interconnected pathways could contribute to this association. Firstly, IR stimulates the secretion of insulin, and hyperinsulinemia leads to increased BMD. Insulin acts as a promoter of osteoblast proliferation and an inhibitor of osteoclast activity, thus playing an anabolic role in bone. In the state of IR, the body compensates for resistance in skeletal muscle, adipose tissue, and liver by increasing insulin secretion, leading to hyperinsulinemia. This elevated insulin level further boosts bone mass^31^. Secondly, IR may also influence bone metabolism through its impact on inflammatory responses and estrogen levels. Inflammatory mediators such as TNF-α, IL-1, and IL-9 have been shown to promote osteoclasts differentiation or inhibit the osteogenic differentiation of bone marrow mesenchymal stem cells via the activation of the RANKL/RANK/OPG and Wnt signaling pathways^32^. Estrogen exerts a direct influence on osteocytes, osteoblasts, and osteoclasts, while also inhibiting osteoclast activation either directly or through osteoblasts and T-cells. The ultimate effect of estrogen on the skeletal system is a reduction in bone remodeling and resorption, while simultaneously preserving bone formation^33^. Finally, the relationship between IR and bone metabolism is bidirectional, suggesting the potential for a cycle: IR impacts bone metabolism, and in turn, the state of bone health influences glucose metabolism^34–38^.

Furthermore, our study revealed thresholds between TyG, TyG-related indices and total BMD. As TyG exceeds the threshold, the regression coefficient increases. Conversely, when TyG-related indices exceed their thresholds, the regression coefficient decreases. The discrepancy may be caused by two factors. On the one hand, as IR progresses, insulin sensitivity significantly decreases, resulting in more insulin release^39^. Insulin is a critical anabolic hormone in osteoblasts^40^. Insulin action in osteoblasts stimulates mitosis, inhibits apoptosis, and prevents hyperglycemia's deleterious effects on bone formation^40,41^. Research have demonstrated that the stimulation of insulin receptors promotes proliferation and differentiation of osteoblasts in immature mice^42^. Mature osteoblasts in culture also express insulin receptors^43^. Osteoblasts are affected by insulin through multiple signaling pathways, thus de-repressing proliferation^44^, blocking the pro-apoptotic effect^45,46^, and promoting anabolism^47^. On the other hand, the involvement of obesity in IR prevents IGF-1 from binding to insulin receptors on osteoblasts, leading to dysfunctional insulin signaling, which negatively affects bone remodeling^48–50^. Obesity is also associated with the loss of Dock 7 protein and silence of Thy-1 expression, which results in higher bone resorption and higher levels of adipogenesis, leading to impaired bone formation^48,51–53^.

In addition to the threshold effect, TyG-BMI and TyG-WC had a saturation effect on total BMD with saturation thresholds of 314.177 and 1022.0428. Saturation might be caused by genetic determinism and co-leadership interactions. Early in life, bone growth trajectories and peak bone mass were determined^54,55^, which might account for the plateau in BMD values observed in adults after a limited increase. Pocock et al. explored the influence of genetics on BMD in 38 pairs of monozygotic twins and 27 pairs of dizygotic twins, revealing that genetics played a pivotal role in adult bone mass^56^. It was determined that genetic factors accounted for 75% of the variance in BMD, regardless of gender^57,58^. Additionally, acquired environmental factors such as calcium intake, estrogen intake, and physical exercise also had limitations in terms of increasing adult BMD^59–61^. Furthermore, bone mass was also dominated by a complex interplay of multiple factors. Research has demonstrated a connection between adipose and bone tissues within the body, with numerous bioactive molecules maintaining bone homeostasis^62^. This bone-adipose axis may offer an explanation for the observed saturation effects. It has been found that adipose and bone tissues share a common stem cell precursor and are in a competitive relationship, with excessive fat accumulation resulting in decreased bone mass^63,64^. Meanwhile, excessive fat accumulation has been linked to decreased bone mass, as confirmed by animal models on high-fat diets^65–67^. According to other studies, adipose tissue also secretes inflammatory mediators such as interleukin-1 and tumor necrosis factor-α^68^, which inhibit BMD via the RANK/RANKL/OPG pathway^69^. In summary, the saturation effects observed in TyG-BMI and TyG-WC could be attributable to a variety of factors. Despite these findings, the existing body of direct experimental evidence remains limited, necessitating further in-depth research.

Indeed, excessive IR and obesity were detrimental to the entire population. Firstly, excessive IR and obesity were associated with a number of bone-related disorders, such as increased bone fragility^70,71^ and increased risk of fractures^19,30^. In addition, IR and obesity can lead to a variety of chronic diseases and complications, such as cardiovascular disease^72,73^, T2DM^74,75^, gallbladder disease^76,77^ and fatty liver^78,79^. More seriously, high IR and excessive obesity might increase cancer risk^80,81^ and cancer-related mortality^82,83^. Therefore, we believe that maintaining TyG and TyG-related indices at reasonable values might contribute to maintaining optimal BMD and reducing the risk of related diseases and complications.

In subgroup analysis, the racial subgroup showed significant differences in the effects of TyG and TyG-related indices on BMD, which might indicate racial differences in bone and mineral metabolism. This may be due to differences in insulin sensitivity and the relationship to triglyceride concentration among the different race/ethnic groups. According to one research^84^, African Americans and non-Hispanic Whites have greater insulin sensitivity. Moreover, African Americans have lower triglyceride levels than non-Hispanic Whites at a given level of IR. Intriguingly, a divergent study with a similar focus on adults, highlighting a negative correlation between TyG and BMD^85^, introduces a notable contrast to our findings. It's crucial to acknowledge that our study included children but excluded postmenopausal females, potentially creating unique subsets within age and gender subgroups. In addition, gender had a significant effect on BMD related to TyG-related indices. This may be related to estrogen, which is an important regulator of body weight, body fat distribution and IRn^86–89^. Given these intriguing disparities observed across racial and gender subgroups, further investigation is warranted. A thorough exploration of the underlying mechanisms driving these differences could offer valuable insights into the complex interplay between insulin resistance and bone health across diverse populations.

In summary, this was a successful large cross-sectional study that revealed a positive association between IR and BMD through TyG and TyG-related indices. Primarily, the utilization of a nationally representative cohort ensured that the results summarized the heterogeneity of the overwhelming majority of demographics in the USA. Furthermore, the large sample size enabled us to conduct subgroup analyses, classifying participants by race, thereby enhancing the generalizability of our findings. However, several limitations of this study merit cautious interpretation of the conclusions. Firstly, the cross-sectional design of the study precludes the establishment of a definitive causal link between TyG, TyG-related indices and BMD. Moreover, database constraints meant that comprehensive data on participants' lifestyle, dietary habits and bone metabolism indices, which might have impacted BMD. The inability to gather BMD data from various skeletons prevented the investigation of the consistency of the outcomes across skeletons. Finally, the absence of fracture data in our database meant we could not determine whether participants with lower TyG or TyG-related indices had a higher incidence of fractures compared to the general population.

## Conclusion

This study revealed a positive association between TyG, TyG-related indices and total BMD. There was also a saturation phenomenon between TyG-BMI, TyG-WC and total BMD. TyG and TyG-related indices can serve as effective indicators for the prevention and treatment of OP. Maintaining optimal levels of TyG and TyG-related indices is critical for managing bone metabolism effectively.

### Supplementary Information

Supplementary Information.

## Data Availability

The survey data are publicly available on the Internet for data users and researchers throughout the world http://www.cdc.gov/nchs/nhanes/.

## Abbreviations

ALP: Alkaline phosphatase
BMD: Bone mineral density
BMI: Body mass index
BUN: Blood urea nitrogen
CPK: Creatine phosphokinase
DXA: Dual-energy X-ray absorption
Family PIR: Ratio of family income to poverty
HDL-C: High-density lipoprotein cholesterol
HECT: Hyperinsulinemic-euglycemic clamp
HOMA-IR: Homeostasis model assessment of insulin resistance
IR: Insulin resistance
LDL-C: Low-density lipoprotein cholesterol
MetS: Metabolic syndrome
NHANES: National Health and Nutrition Examination study database
OP: Osteoporosis
T2DM: Type 2 diabetes mellitus
TyG: Triglyceride glucose index
TyG-BMI: Triglyceride glucose-body mass index
TyG-WC: Triglyceride glucose-waist circumference
TyG-WHtR: Triglyceride glucose-waist-to-height ratio
WC: Waist circumference
WHtR: Waist-to-height ratio
