# Supplementary material for: Association between triglyceride glucose index and total bone mineral density: a cross-sectional study from NHANES 2011–2018
Source: Sci Rep. 2024 Feb 20;14:4208. doi: 10.1038/s41598-024-54192-9 (PMC10879154; doi:10.1038/s41598-024-54192-9)
Supplement: Supplementary file 1 — Supplementary Information. [file 41598_2024_54192_MOESM1_ESM.pdf]

Association between triglyceride glucose index and total bone mineral density: a cross-sectional study from NHANES 2011-2018

Ningsheng Tian[1†], Shuai Chen[1†], Huawei Han[1], Jie Jin[1], Zhiwei Li[1*]

[1]Department of Orthopaedics, The Second Affiliated Hospital of Nanjing University of Chinese Medicine, Nanjing, China

†Ningsheng Tian and Shuai Chen contributed equally to this work

***Correspondence:**
Zhiwei Li
Department of Orthopaedics, The Second Affiliated Hospital of Nanjing University of Chinese Medicine, No.23, Nanhu Road, Jianye District, Nanjing 210017, Jiangsu Province, People's Republic of China. E-mail: 039216235@njucm.edu.cn.

Supplementary Table S1: The used NHANES variable code

	**NHANES variable code**	**Remarks**
Age (years)	RIDAGEYR	inclusion
Gender (%)	RIAGENDR	inclusion
Race/ethnicity (%)	RIDRETH1	inclusion
Education level (%)	DMDEDUC2+DMDEDUC3	inclusion
Family PIR	INDFMPIR	inclusion
Weight (kg)	BMXWT	inclusion
Height (cm)	BMXHT	inclusion
BMI (kg/cm^2)	BMXBMI	inclusion
WC (cm)	BMXWAIST	inclusion
Fasting glucose (mg/dL)	LBXGLU	inclusion
Fasting triglyceride (mg/dL)	LBXTR	inclusion
ALP (IU/L)	LBXSAPSI	inclusion
BUN (mg/dL)	LBXSBU	inclusion
CPK (IU/L)	LBXSCK	inclusion
Creatinine (mg/dL)	LBXSCR	inclusion
Phosphorus (mg/dL)	LBXSPH	inclusion
Total calcium (mg/dL)	LBXSCA	inclusion
Uric acid (mg/dL)	LBXSUA	inclusion
Total bilirubin (mg/dL)	LBXSTB	inclusion
Glycohemoglobin (%)	LBXGH	inclusion
Total cholesterol (mg/dL)	LBXTC	inclusion
HDL-C (mg/dL)	LBDHDD	inclusion
LDL-C (mg/dL)	LBDLDL	inclusion
25OHD2 + 25OHD3 (nmol/L)	LBXVIDMS	inclusion
Total BMD (g/cm^2)	DXDTOBMD	inclusion
Case with thyroid disease	KIQ022	Exclude KIQ022-1
Case with cancer or malignancy	MCQ220	Exclude MCQ220-1
Case with nephropathy	MCQ160m	Exclude MCQ160m-1
Case with postmenopausal	RHQ031	Exclude RHQ031-2

Supplementary Table S2: Basic characteristics of the research population based on Fasting insulin quartiles.

**Fasting insulin (µU/mL)**	**Quartile 1 (0.14-6.23)**	**Quartile 2 (6.24-9.49)**	**Quartile 3 (9.50-15.30)**	**Quartile 4 (15.31-195.20)**	***P*-value**
TYG	8.03 ± 0.56	8.30 ± 0.60	8.47 ± 0.60	8.70 ± 0.59	<0.0001
TYGBMI	190.78 ± 38.00	215.00 ± 47.01	241.25 ± 57.46	289.83 ± 72.07	<0.0001
TYGWHTR	4.00 ± 0.64	4.41 ± 0.79	4.83 ± 0.91	5.57 ± 1.04	<0.0001
TYGWC	679.53 ± 114.85	746.61 ± 142.95	815.46 ± 162.81	941.39 ± 182.62	<0.0001

Supplementary Table S3: Basic characteristics of the research population based on HOMA-IR quartiles.

HOMA-IR	Quartile 1 (0.005-0.296)	Quartile 2 (0.297-0.467)	Quartile 3 (0.468-0.784)	Quartile 4 (0.785-23.186)	*P*-value
TYG	8.00 ± 0.53	8.26 ± 0.57	8.47 ± 0.57	8.78 ± 0.62	<0.0001
TYGBMI	189.89 ± 37.21	211.95 ± 45.90	240.27 ± 53.67	294.06 ± 70.89	<0.0001
TYGWHTR	3.98 ± 0.62	4.35 ± 0.75	4.82 ± 0.85	5.65 ± 1.02	<0.0001
TYGWC	674.69 ± 110.66	735.81 ± 136.17	814.22 ± 151.31	957.14 ± 180.02	<0.0001

Supplementary Table S4: Subgroup analysis between TyG and total BMD.

	Model 3<br>β (95%CI), *P*-value	Model 4<br>β (95%CI), *P*-value
**Stratified by gender**		
Male	0.0101 (-0.0055, 0.0256)<br>0.2049	0.0093 (-0.0063, 0.0248)<br>0.2420
Female	0.0182 (0.0006, 0.0357)<br>0.0430	0.0168 (-0.0008, 0.0343)<br>0.0613
P for interaction	0.3478	0.2571
**Stratified by race**		
Mexican American	0.0366 (0.0090, 0.0642)<br>0.0096	0.0372 (0.0097, 0.0647)<br>0.0082
Non-Hispanic White	0.0123 (-0.0080, 0.0326)<br>0.2368	0.0116 (-0.0087, 0.0318)<br>0.2640
Non-Hispanic Black	0.0298 (0.0054, 0.0542)<br>0.0168	0.0242 (0.0000, 0.0485)<br>0.0503
Other race/ethnicity	-0.0056 (-0.0265, 0.0154)<br>0.6031	-0.0066 (-0.0277, 0.0145)<br>0.5399
P for interaction	0.0198	0.0186

Model 3: age, gender, race, education level, family PIR, ALP, BUN, CPK, creatinine, phosphorus, total calcium, uric acid, total bilirubin, glycohemoglobin, total cholesterol, HDL-C, LDL-C and 25OHD2 + 25OHD3 were adjusted. Model 4: age, gender, race, education level, ALP, BUN, CPK, creatinine, phosphorus, total calcium, uric acid, total bilirubin, glycohemoglobin, total cholesterol, HDL-C, LDL-C and 25OHD2 + 25OHD3 were adjusted.

Supplementary Table S5: Subgroup analysis between TyG-BMI and total BMD.

	Model 3<br>β (95%CI), *P*-value	Model 4<br>β (95%CI), *P*-value

**Stratified by gender**		
Male	0.0003 (0.0002, 0.0004) <0.0001	0.0003 (0.0002, 0.0004) <0.0001
Female	0.0004 (0.0003, 0.0005) <0.0001	0.0004 (0.0003, 0.0005) <0.0001
P for interaction	0.0741	0.0732
**Stratified by race**		
Mexican American	0.0004 (0.0003, 0.0006) <0.0001	0.0004 (0.0003, 0.0005) <0.0001
Non-Hispanic White	0.0003 (0.0002, 0.0004) <0.0001	0.0003 (0.0002, 0.0004) <0.0001
Non-Hispanic Black	0.0003 (0.0002, 0.0004) <0.0001	0.0003 (0.0002, 0.0004) <0.0001
Other race/ethnicity	0.0005 (0.0004, 0.0006) <0.0001	0.0005 (0.0004, 0.0006) <0.0001
P for interaction	0.0002	0.0022

Model 3: age, gender, race, education level, family PIR, ALP, BUN, CPK, creatinine, phosphorus, total calcium, uric acid, total bilirubin, glycohemoglobin, total cholesterol, HDL-C, LDL-C and 25OHD2 + 25OHD3 were adjusted. Model 4: age, gender, race, education level, ALP, BUN, CPK, creatinine, phosphorus, total calcium, uric acid, total bilirubin, glycohemoglobin, total cholesterol, HDL-C, LDL-C and 25OHD2 + 25OHD3 were adjusted.

Supplementary Table S6: Subgroup analysis between TyG-WHtR and total BMD.

	**Model 3** **β (95%CI), *P*-value**	**Model 4** **β (95%CI), *P*-value**
**Stratified by gender**		
Male	0.0036 (-0.0019, 0.0091) 0.1962	0.0030 (-0.0024, 0.0085) 0.2773
Female	0.0191 (0.0141, 0.0241) <0.0001	0.0191 (0.0141, 0.0242) <0.0001
P for interaction	0.0002	0.0002
**Stratified by race**		
Mexican American	0.0192 (0.0103, 0.0282) <0.0001	0.0184 (0.0095, 0.0273) <0.0001
Non-Hispanic White	0.0048 (-0.0018, 0.0114) 0.1557	0.0041 (-0.0026, 0.0107) 0.2295

Non-Hispanic Black	0.0117 (0.0032, 0.0202)<br>0.0070	0.0101 (0.0017, 0.0186)<br>0.0193
Other race/ethnicity	0.0232 (0.0160, 0.0304)<br><0.0001	0.0221 (0.0148, 0.0294)<br><0.0001
P for interaction	0.0036	0.0099

Model 3: age, gender, race, education level, family PIR, ALP, BUN, CPK, creatinine, phosphorus, total calcium, uric acid, total bilirubin, glycohemoglobin, total cholesterol, HDL-C, LDL-C and 25OHD2 + 25OHD3 were adjusted. Model 4: age, gender, race, education level, ALP, BUN, CPK, creatinine, phosphorus, total calcium, uric acid, total bilirubin, glycohemoglobin, total cholesterol, HDL-C, LDL-C and 25OHD2 + 25OHD3 were adjusted.

Supplementary Table S7: Subgroup analysis between TyG-WC and total BMD.

	**Model 3**<br>**β (95%CI), *P*-value**	**Model 4**<br>**β (95%CI), *P*-value**
**Stratified by gender**		
Male	0.0001 (0.0001, 0.0001)<br><0.0001	0.0001 (0.0001, 0.0001)<br><0.0001
Female	0.0002 (0.0001, 0.0002)<br><0.0001	0.0002 (0.0001, 0.0002)<br><0.0001
P for interaction	0.0027	0.0036
**Stratified by race**		
Mexican American	0.0002 (0.0001, 0.0002)<br><0.0001	0.0002 (0.0001, 0.0002)<br><0.0001
Non-Hispanic White	0.0001 (0.0000, 0.0001)<br><0.0001	0.0001 (0.0000, 0.0001)<br><0.0001
Non-Hispanic Black	0.0001 (0.0001, 0.0001)<br><0.0001	0.0001 (0.0001, 0.0001)<br><0.0001
Other race/ethnicity	0.0002 (0.0001, 0.0002)<br><0.0001	0.0002 (0.0001, 0.0002)<br><0.0001
P for interaction	0.0002	0.0011

Model 3: age, gender, race, education level, family PIR, ALP, BUN, CPK, creatinine, phosphorus, total calcium, uric acid, total bilirubin, glycohemoglobin, total cholesterol, HDL-C, LDL-C and 25OHD2 + 25OHD3 were adjusted. Model 4: age, gender, race, education level, ALP, BUN, CPK, creatinine, phosphorus, total calcium, uric acid, total bilirubin, glycohemoglobin, total cholesterol, HDL-C, LDL-C and 25OHD2 + 25OHD3 were adjusted.
